# Effects of total replacement of corn silage with sorghum silage on milk yield, composition, and quality

**DOI:** 10.1186/s40104-017-0146-8

**Published:** 2017-01-31

**Authors:** M. Cattani, N. Guzzo, R. Mantovani, L. Bailoni

**Affiliations:** 1 Department of Comparative Biomedicine and Food Science (BCA), University of Padova, Viale dell’Università 16, 35020 Legnaro, PD Italy; 2 Department of Agronomy Food Natural resources Animals and Environment (DAFNAE), University of Padova, Viale dell’Università 16, 35020 Legnaro, PD Italy

**Keywords:** Dairy cows, Forage sorghum silage, Mean particle size, Milk coagulation properties, Milk fatty acid profile

## Abstract

**Background:**

In the last years, difficulties occurring in corn cultivation (i.e., groundwater shortages, mycotoxin contamination) have been forcing dairy farmers to consider alternative silages. Some experiments conducted on lactating cows have proven that the total replacement of corn silage with sorghum silage did not reduce milk yield. However, this kind of substitution involves supplementing sorghum-based diets with grains, to compensate for the lower starch content of sorghum silage compared to corn silage. Change of silage type and inclusion of starch sources in the diet would influence rumen fermentations, with possible effects on milk composition (i.e., fatty acid profile) and coagulation properties. A worsening of milk coagulation properties would have a negative economic impact in Italy, where most of the milk produced is processed into cheese.

This study was designed to compare milk composition and quality, with emphasis on fatty acid profile and coagulation properties, in dairy cows fed two diets based on corn or sorghum silage.

**Results:**

The sorghum diet reduced milk yield (*P* = 0.043) but not 4% fat corrected milk (*P* = 0.85). Feeding sorghum silage did not influence milk contents of protein (*P* = 0.07) and lactose (*P* = 0.65), and increased fat content (*P* = 0.024). No differences emerged for milk concentrations of saturated (*P* = 0.61) and monounsaturated fatty acids (*P* = 0.50), whereas polyunsaturated fatty acids were lower (*P* < 0.001) for the sorghum diet. Concentrations of n-6 (*P* < 0.001) and n-3 fatty acids (*P* = 0.017) were lower in milk of cows fed the sorghum diet. Milk coagulation properties did not differ between the two diets, except the “a30” (the curd firmness, expressed in mm, 30 min after rennet addition), that was lower (*P* = 0.042) for the sorghum diet.

**Conclusions:**

Feeding a forage sorghum silage, properly supplemented with corn meal, as total replacement of corn silage maintained milk composition and did not influence negatively milk coagulation properties, which have a great economic relevance for the Italian dairy industry. Thus, silages obtained from forage sorghums could have a potential as substitute of corn silages in dairy cow diets.

## Background

Corn silage is the main ingredient of diets fed to lactating cows in the farms of the Po Valley (North-Italy), with exception of those producing milk processed into some Protected Denomination of Origin cheeses (i.e., Parmigiano-Reggiano). However, because of some difficulties occurring in corn cultivation (i.e., groundwater shortages, plant attack by specific parasites, mycotoxin contamination), in the last few years dairy farmers have been considering the use of alternative forages for ensiling. Experiments conducted on lactating cows have proven that the total replacement of corn silage with sorghum silage did not affect milk yield [1–4]. However, in some studies such results were obtained through the supplementation of sorghum diets with starch sources (i.e., corn meal), to compensate for the lower starch content of sorghum silage compared to corn silage [3, 4]. There is evidence from literature that the change of silage type and the inclusion of starch sources in the diet would influence rumen fermentation patterns, with effects on composition of milk fat [5] and milk protein [6]. In turn, a modification of protein fractions would affect some milk coagulation properties (MCP) as renneting time and curd firmness [7]. To our knowledge, no attempts have been made to explore whether the total replacement of corn silage with sorghum silage could influence milk fatty acids (FA) profile and MCP. These milk properties have a great economic relevance in Italy, where about 50% of cow milk is processed into Protected Denomination of Origin (PDO) cheeses, even if some specifications (i.e. that of Parmigiano-Reggiano cheese) do not allow the use of silages in dairy cow diets.

In this framework, this study was designed to compare milk yield, composition, and quality, with particular emphasis on milk FA profile and MCP, in dairy cows fed two experimental diets containing silages obtained from corn or forage sorghum.

## Methods

### Corn and forage sorghum cultivation, harvest, and ensiling

Corn plants (hybrid Kayras, FAO class 600; KWS Italia Spa, Monselice, Italy) were sown on 29 May, 2014 on a soil that previously hosted alfalfa (*Medicago sativa*). The soil belonged to a farmhouse located in San Martino di Lupari, Province of Padova, latitude 45.3°N, longitude 11.5°E; elevation: 40 m above sea level. The corn was fertilized with 200 units of Nitrogen, 140 units of Phosphorous, and 180 units of Potassium/hectare, and weed control was applied with 1.5 L/hectare of herbicide Adengo (Bayer CropScience S.r.l. Milano, Italy). Corn was harvested at two-thirds milk line stage of maturity on 30 September, 2014. Plants of forage sorghum (hybrid Hannibal; KWS Italia Spa, Monselice, Italy) were sown on an adjacent field in the same farmhouse as above on 17 June, 2014 on a soil that previously hosted barley. The sorghum was fertilized with 160 units of Nitrogen, 80 units of Phosphorous, and 100 units of Potassium/hectare, and weed control was applied with 0.3 kg/hectare of herbicide Casper (Syngenta Italia S.p.A., Milano, Italy). Sorghum was harvested at the beginning of the early bloom stage, on 30 September, 2014. No irrigation was needed for either forages due to the favorable climate situation. For both corn and sorghum a forage harvester with 8 line-head for chopping (New Holland Agriculture, Turin Italy) was used. Corn plants were harvested with knives adjusted to a 13-mm theoretical length of cut and processed with a 3-mm roller clearance. Sorghum plants were harvested with knives adjusted to a 20-mm theoretical length of cut, without any processing. After harvest, corn and forage sorghum were ensiled into bunker silos without inoculation and covered with nylon film for 60 d. Sample of silages (n = 4 per corn and sorghum) were obtained collecting about 3 kg of silage from 4 different points in the front of each bunker silos. The chemical composition of the two silages is given in Table 1.

**Table 1:** Descriptive statistics for nutrient composition, fatty acid profile, and particle size of the two silages used in the study

Component (g/kg DM unless noted)	Corn silage		Sorghum silage	
	Mean	SEM	Mean	SEM
Nutrients				
DM as fed	331.9	4.8	222.7	0.8
CP	76.8	0.9	73.7	1.9
Fat	29.0	1.2	24.7	0.7
Ash	43.2	6.0	79.3	0.4
NDF	348.7	10.0	711.3	14.8
ADF	197.4	5.7	459.9	11.5
ADL	29.5	1.2	73.3	1.9
Starch	351.9	20.2	25.7	2.4
NSC^a^	502.4	10.6	111.9	15.8
NE_L_^b^, Mcal/kg DM	1.82	0.02	0.92	0.04
pH	3.68	0.02	4.08	0.07
Fatty acid profile				
SFA^c^, % of total FA^d^				
C16:0	14.4	0.1	22.6	0.5
C18:0	2.31	0.03	2.77	0.21
Total SFA	19.7	0.4	34.2	0.9
MUFA^e^, % of total FA				
C18:1 n-9	15.3	2.0	5.2	1.1
Total MUFA	17.9	2.0	9.1	1.0
PUFA^f^, % of total FA				
C18:2 n-6	54.4	1.5	22.6	1.2
C18:3 n-3	7.0	0.3	32.5	1.9
Total PUFA	62.4	1.7	56.7	0.8
n-6, % of total FA	54.5	1.5	22.9	1.2
n-3, % of total FA	7.2	0.4	32.6	1.9
Particle size fraction, % retained (as-fed basis)				
> 19 mm	4.3	0.8	4.6	0.9
> 8 to 19 mm	68.0	1.4	80.7	0.9
< 8 mm	27.7	1.2	14.7	0.3
pef^g^	0.72	0.01	0.85	0.01
peNDF^h^, % of DM	24.6	0.6	62.1	0.4
Mean particle size^i^, mm	6.2	0.2	8.7	0.1

^a^ *NSC* non-structural carbohydrates, calculated as: 1000 – (CP + Fat + Ash + NDF), ^b^ *NE*_L_ calculated according to NRC (2001 [8]), ^c^ *SFA* saturated fatty acids, ^d^ *FA* fatty acids, ^e^ *MUFA* monounsaturated fatty acids, ^f^ *PUFA* polyunsaturated fatty acids, ^g^ *pef* physical effectiveness factor, calculated as sum of the proportion of feed particles retained on sieves with openings of 19 and 8 mm, ^h^ *peNDF* physically effective NDF, calculated as pef multiplied by the corresponding NDF content, ^i^Mean particle size = calculated according to ASABE (2007 [17])

### Treatments, animals and experimental design

All experimental procedures were carried out according to Italian law on animal care (Legislative Decree No. 26 of March 14, 2014). Eighteen Holstein-Friesian dairy cows (DIM: 146 ± 96 d; parity: 2.0 ± 1.2; milk yield: 28.4 ± 6.3 kg/d), housed in a tie-stall commercial dairy farm belonging to the farmhouse described above (San Martino di Lupari, Padova province), were used. Animals were randomly assigned to 2 groups subjected to 2 treatment sequences in a cross-over experimental design of 28-d periods. Each experimental period was preceded by a 10-d preliminary period, thus the whole experiment lasted 76 d. Each group received a total mixed ration (TMR) based on corn silage (CS diet) or forage sorghum silage (SS diet). Two silages were included in different proportions (295 and 195 g/kg DM for CS and SS, respectively), according to their chemical composition. The SS diet contained an amount of corn meal that was almost double compared to the CS diet (254 vs. 142 g/kg DM), in order to compensate for the lower starch content of forage sorghum silage compared to corn silage. The two TMR were prepared using a total mixer wagon. During preparation of TMR, the two experimental diets were only mixed, without a further chopping of silages. The diets were formulated to be isoenergetic and isonitrogenous using the Plurimix system® (Fabermatica Sas, Ostiano, Cremona, Italy), and according to NRC system [8] considering a predicted milk yield of about 30 kg/d and a feed intake of about 25 kg/d per cow. The chemical composition of the experimental diets is given in Table 2. The forage NDF contents resulted 182 and 218 g/kg DM for CS and SS, respectively [8] (data not shown). During the trial the cows had free access to water and they were fed once daily. Cows were milked twice per day (at 0700 h and 1900 h). Milking procedure occurred at stall using a milking pipeline system.

**Table 2:** Ingredients ad descriptive statistics for nutrient composition, fatty acid profile, and particle size of the two experimental diets

Component (g/kg DM unless noted)	Corn silage diet		Sorghum silage diet	
	Mean	SEM	Mean	SEM
Ingredients				
Corn silage	295	-	-	-
Sorghum silage	-	-	195	-
Alfalfa hay	172	-	170	-
Corn meal	142	-	254	-
Soybean meal	87	-	85	-
Barley meal	65	-	63	-
Cottonseeds	65	-	63	-
Dry sugar beet pulp	53	-	52	-
Distillers dried grains with solubles	44	-	43	-
Wheat straw	44	-	43	-
Soybean hulls	30	-	30	-
Urea	2	-	2	-
Nutrients				
DM, % fresh matter	539.2	2.1	158.9	0.2
CP	141.6	3.3	142.6	2.7
Fat	38.3	2.4	33.1	1.1
Ash	67.7	0.8	69.4	2.4
NDF	365.1	2.9	397.0	9.1
ADF	218.8	2.5	238.4	3.9
ADL	41.9	1.5	47.7	1.0
Starch	229.5	8.2	202.3	10.7
NSC^a^	387.4	0.6	357.9	9.3
NE_L_^b^, Mcal/kg DM	1.61	0.02	1.59	0.02
Fatty acid profile				
SFA^c^, % of total FA^d^				
C16:0	18.7	0.2	19.3	0.1
C18:0	2.48	0.03	2.28	0.02
Total SFA	24.6	0.3	24.8	0.1
MUFA^e^, % of total FA				
C18:1 n-9	18.8	0.3	19.3	0.3
Total MUFA	21.6	0.3	21.7	0.2
PUFA^f^, % of total FA				
C18:2 n-6	49.3	0.2	48.6	0.7
C18:3 n-3	4.0	0.2	4.2	0.4
Total PUFA	53.8	0.1	53.4	0.3
n-6, % of total FA	49.4	0.2	48.6	0.7
n-3, % of total FA	4.0	0.2	4.2	0.4
Particle size fraction, % retained (as-fed basis)				
> 19 mm	5.7	1.4	3.8	0.6
> 8 to 19 mm	27.8	0.7	33.7	0.9
< 8 mm	66.5	1.4	62.5	0.8
pef^g^	0.34	0.01	0.37	0.01
peNDF^h^, % of DM	12.3	0.5	15.2	0.5
Mean particle size^i^, mm	2.2	0.1	2.4	0.1

^a^ *NSC* non-structural carbohydrates, calculated as: 1000 – (CP + Fat + Ash + NDF), ^b^ *NE*_*L*_ calculated according to NRC (2001 [8]), ^c^ *SFA* saturated fatty acids,^d^ *FA* fatty acids, ^e^ *MUFA* monounsaturated fatty acids, ^f^ *PUFA* polyunsaturated fatty acids, ^g^ *pef* physical effectiveness factor, calculated as sum of the proportion of feed particles retained on sieves with openings of 19 and 8 mm, ^h^ *peNDF* physically effective NDF, calculated as pef multiplied by the corresponding NDF content, ^i^Mean particle size = calculated according to ASABE (2007 [17])

### Data and sample collection

The amount of diet distributed in the manger of each experimental group was measured daily and recorded by the weighing station of the mixer wagon. The orts were daily collected and analyzed for DM in order to obtain the daily DM intake (DMI), on a group basis, as difference between consumed and residual DM. Individual milk yield was recorded and individual milk samples (50 mL/each) from the morning milking only were collected from each cow to be analyzed for composition, FA profile, and MCP (n = 2 at about d 15 and d 28 of each period for a total of 4 samples per cow). Representative samples (about 3 kg as fed) of the two silages and of the two diets were collected to be analyzed for chemical composition and particle size distribution. Feed and diet samples were collected at the beginning and at the end of each period (n = 4 samples for each silage and diet).

### Sample analysis

Samples of silages and diets were analyzed in duplicate for DM (DM; # 934.01; [9]), N (# 976.05; [9]), lipids (# 920.29; [9]) and ash (# 942.05; [9]). The NDF content was measured using α-amylase and sodium sulphite, with the Ankom^220^ Fiber Analyzer (Ankom Technology®, Macedon, NY, USA). The ADF, inclusive of residual ash, and ADL contents were sequentially determined according to [10]. Starch was analyzed by high-performance liquid chromatography, following the quickly and more precise to conventional assay method suggested by [11]. The FA profile of silages and diets was determined according to procedure suggested by [12].

Fat, protein, and lactose contents of the milk were analyzed using the FIL-IDF procedure [13] by MilkoScan™ FT1 apparatus (Foss Electric, DK-3400, Hillerød, Denmark). Milk urea nitrogen was measured automatically by the conduct metric-enzymatic method (CL 10 micro analyser, Eurochem, Roma, Italy). Somatic cell count was performed using a Fossomatic^TM^ 5000 (Foss Electric, DK-3400, Hillerød, Denmark) according to the standard FIL-IDF148a [14], and transformed in logarithmic terms using the following equation: SCS = 3 + ln_2_ (somatic cell count × 10^−5^). Milk lipids were extracted according to the procedures described by [15]. Fatty acid methyl esters were analyzed using a two-dimensional gas-chromatography instrument (Agilent 7890A, Agilent Technologies, Milan, Italy) equipped with a modulator (Agilent G3486 A CFT), an automatic sampler (Agilent 7693), a flame-ionization detector connected to a chromatography software (Agilent Chem Station), and two columns in series, to separate and identify each FA on a 2-dimensional basis [15]. The first column was a 75 m × 180 μm (internal diameter) × 0.14 μm film thickness column (23348U, Supelco, Bellefonte, PA) and used H_2_ as carrier gas (flow of 0.22 mL/min). The second was a 3.8 m × 250 μm (internal diameter) × 0.25 μm film thickness column (J&W 19091-L431, Agilent Technologies) and used H_2_ as carrier gas (flow of 22 mL/min). A Computerized Renneting Meter (CRM-48, Polo Trade, Monselice, Italy) was used to determine MCP within 5 h after collection of samples. Milk samples (10 mL) were preheated at 35°C, and 200 μL of rennet (NATUREN TM STANDARD 215, Hansen 215 IMCU/mL, Pacovis Amrein AG, Bern, Switzerland) were diluted to 1.2% (v/v) in distilled water and then added to the milk. This analysis provided measurements of rennet coagulation time (RCT; the time occurring from addition of rennet to the beginning of coagulation), k20 (the time to observe a curd firmness of 20 mm after the rennet was added to the sample), and a30 (curd firmness 30 min after rennet addition). Measures of pH (pH-Burette 24, Crison) were conducted before measuring MCP.

The particle size distribution of silages and diets was determined by dry sieving using a Penn State Particle Separator equipped with three screens (diameter openings = 19 mm, 8 mm, plus the bottom pan). Approximately 250 g of feed sample was placed on the upper screen of the separator and sieved according to the procedure described by [16].

### Calculations and statistical analysis

Mean particle size of silages and diets was calculated according to [17]. The physical effectiveness factor (pef; ranging from 0 to 1) of silages and diets was computed as the sum of the proportion of feed particles retained on sieves with openings of 19 and 8 mm [18]. The content of physically effective NDF (peNDF; % of DM) of silages and diets was calculated by multiplying the pef by the corresponding NDF content [18]. Yield of solids-corrected milk (SCM) was computed according to [19]. Feed efficiency of cows, intended as the ability to convert feed to milk, was calculated as the ratio between the SCM and the DMI.

All individual data were preliminarily analyzed using the TTSET procedure of [20] to investigate possible carryover effects due to the sequence. Because of the washout interval of 10 d between the two experimental periods, no carryover was detected. Data were then analyzed by a MIXED procedure of [20] accounting for the fixed effects of diet (CS and SS), period (first and second period), and sequence of treatment (CS-SS or SS-CS sequence), and the random effect of cow. A simple one way ANOVA by a GLM procedure of [20] was carried out for the dry matter intake accounting for the diet effect only.

## Results

### Chemical composition and mean particle size of silages and diets

Compared to the corn silage, the sorghum silage showed, as expected, a lower DM content (223 vs. 332 g/kg; Table 1). On the contrary, the protein and lipid contents were similar for the two silages. As expected, levels of fibrous fractions (NDF, ADF, and ADL) resulted greater for the forage sorghum silage compared to the corn silage. Consequently, the sorghum silage showed a lower content of NSC (non-structural carbohydrates; 112 vs. 502 g/kg DM) and starch (26 vs. 352 g/kg DM) with respect to the corn silage. Compared to the corn silage, the sorghum silage had a greater proportion of saturated fatty acids (SFA) and n-3 but lower proportions of MUFA and PUFA (Table 1). As regards to the main FA, the sorghum silage showed higher proportions of palmitic and α-linolenic acid and lower proportions of oleic and linoleic acid. Considering the particle size distribution, the sorghum silage had a greater percentage of feed material retained on the screen of 8 mm and a lower percentage retained on the bottom pan in comparison with the corn silage. The sorghum silage had a greater pef (0.85 vs. 0.72) and peNDF (62.1 vs. 24.6% of DM) compared to the corn silage, as a consequence of particle size distribution and greater NDF content, and a greater mean particle size. Compared to the CS diet, the SS diet had a greater NDF content (397 vs. 365 g/kg DM; Table 2) and a lower starch content (202 vs. 229 g/kg DM). The diets did not differ for FA composition, showing comparable contents of SFA, MUFA, and PUFA. As regards to the main FA, the only difference emerged for stearic acid that was lower for the SS diet compared to the CS diet. The SS diet showed a greater percentage of feed particles retained on the screen of 8 mm and a greater value of peNDF with respect to the CS diet (Table 2). However, the two diets did not differ in terms of pef and mean particle size.

### Dry matter intake, milk yield, milk composition and coagulation properties, and feed efficiency

The DMI did not differ between the two diets (*P* = 0.88; Table 3). Milk yield (kg/d) was lower (29.8 vs. 31.6 kg/d; *P* = 0.043) for the SS diet, whereas yields of 4% fat corrected milk (*P* = 0.85), fat (*P* = 0.78), protein (*P* = 0.23), and lactose (*P* = 0.07) did not differ between the two diets. The yield of SCM was comparable for the two diets (*P* = 0.59) and resulted 31.6 and 31.0 kg/d, for the CS and the SS diet, respectively. In terms of milk composition, the SS diet resulted in a greater fat content (4.26 vs. 3.98%; *P* = 0.02) and a nominally greater protein content (3.66 vs. 3.55%; *P* = 0.07) compared to the CS diet. Lactose, urea and SCS in milk were not influenced by the diet. Except for the value of a30, that was lower (31.9 vs. 35.5 mm; *P* = 0.04) in the SS diet, milk coagulation properties did not differ between diets (Table 3). Milk samples of cows fed the SS diet showed, on average, a small but significantly greater pH compared to those fed the CS diet (6.84 vs. 6.86, for the CS and SS diet, respectively; *P* = 0.008).

**Table 3:** Least square means and pooled standard error (SE) for DMI, milk yield and composition of cows fed the two experimental diets

Item	Diet		Pooled SE	*P* value
	Corn silage	Sorghum silage		
DMI, kg/d	24.88	24.52	1.44	0.878
Yield, kg/d				
Milk	31.63	29.79	1.97	0.043
4% FCM^a^	31.83	31.54	1.92	0.848
Fat	1.23	1.25	0.07	0.781
Protein	1.11	1.07	0.06	0.229
Lactose	1.54	1.45	0.10	0.067
SCM^b^	31.63	31.02	1.73	0.589
Milk composition, %				
Fat	3.98	4.26	0.16	0.024
Protein	3.55	3.66	0.08	0.065
Lactose	4.85	4.84	0.04	0.634
Urea, mg/L	24.01	25.42	0.52	0.510
SCS^c^, units	3.59	3.65	0.44	0.897
Milk coagulation properties				
RCT^d^, min	19.75	19.76	1.26	0.986
k20^e^, min	6.66	6.50	0.35	0.732
a30^f^, mm	35.45	31.90	2.25	0.042
pH	6.84	6.86	0.02	0.008
Feed efficiency, kg/kg				
SCM:DMI	1.27	1.27	0.07	0.969

^a^ *FCM* milk corrected milk, ^b^ *SCM* solids-corrected milk, calculated according to Tyrrell and Reid (1965 [19]), ^c^ *SCS* somatic cell score, calculated as: SCS = 3 + ln_2_(somatic cell count/100, 000), ^d^ *RCT* rennet coagulation time, ^e^ *k20* time required to reach a curd firmness of 20 mm, ^f^ *a30* curd firmness 30 min after the addition of rennet

Feed efficiency, expressed as the ratio between the SCM and the DMI, was similar for the two groups of cows (*P* = 0.97).

Total concentrations of saturated FA and monounsaturated FA in milk did not differ between the two diets (*P* = 0.61 and *P* = 0.50, respectively; Table 4), whereas that of polyunsaturated FA resulted lower (4.07 vs. 4.58% of total FA; *P* < 0.001) for the SS diet in comparison to the CS diet (Table 4). As a consequence, the ratio between monounsaturated FA and polyunsaturated FA was greater (6.27 vs. 5.51% of total FA; *P* < 0.001) for the SS diet compared to the CS diet. Among saturated FA, concentrations of butyric (C4:0; *P* = 0.003), caprylic (C8:0; *P* = 0.003), palmitic (C16:0; *P* = 0.010), and stearic acid (C18:0; *P* = 0.019) were greater in the milk of cows fed the SS diet; opposite trends were observed for capric (C10:0), and lauric acid (C12:0). Among polyunsaturated FA, concentrations of n-6 and n-3 were lower in milk of cows fed the SS diet (*P* < 0.001 and *P* = 0.017, respectively). The ratio n-6/n-3 also resulted lower (*P* < 0.001) for the SS diet compared to the CS diet. Conjugated linoleic acid isomers (CLA) content, expressed as the sum of the single isomers, was greater (*P* = 0.004) in the milk of cows fed the SS diet.

**Table 4:** Least square means and pooled standard error (SE) for milk fatty acids from cows fed the two experimental diets

Item	Diet		Pooled SE	*P* value
	Corn silage	Sorghum silage		
SFA^a^, % of total FA^b^				
C4:0	3.02	3.24	0.06	0.003
C6:0	2.09	2.08	0.02	0.507
C8:0	1.31	1.21	0.02	0.003
C10:0	3.11	2.71	0.09	<0.001
C12:0	3.69	3.13	0.12	0.080
C14:0	11.90	11.00	0.36	0.062
C16:0	30.76	32.05	0.56	0.010
C18:0	9.93	10.94	0.35	0.019
Others	4.57	4.26	0.11	0.037
Total SFA	70.38	70.60	0.59	0.607
MUFA^c^, % of total FA				
C14:1	0.92	0.87	0.05	0.116
C16:1	1.28	1.26	0.05	0.563
C18:1 n-7	1.87	2.03	0.06	0.018
C18:1 n-9	17.75	18.39	0.50	0.124
Others	3.22	2.77	0.06	<0.001
Total MUFA	25.04	25.32	0.51	0.496
PUFA^d^, % of total FA				
C18:2 n-6	2.48	2.02	0.07	<0.001
C18:2 c9, t11 CLA^e^	0.44	0.52	0.02	0.006
C18:2 t10, c12 CLA	0.01	0.01	0.01	0.396
C18:3 n-3	0.30	0.27	0.01	0.001
C18:3 n-6	0.06	0.05	0.01	0.044
C20:3 n-6	0.15	0.13	0.01	<0.001
C20:4 n-6	0.19	0.16	0.01	<0.001
Others	0.97	0.91	0.03	0.031
Total PUFA	4.58	4.07	0.12	<0.001
SFA/(MUFA + PUFA)	2.40	2.42	0.07	0.714
MUFA/PUFA	5.51	6.27	0.14	<0.001
n-6, % of total FA	2.91	2.39	0.08	<0.001
n-3, % of total FA	0.39	0.36	0.01	0.017
n-6/n-3	7.49	6.73	0.16	<0.001
CLA, % of total FA	0.49	0.58	0.02	0.004

^a^ *SFA* saturated fatty acids, ^b^ *FA* fatty acids, ^c^ *MUFA* monounsaturated fatty acids, ^d^ *PUFA* polyunsaturated fatty acids, ^e^ *CLA* conjugated linoleic acid isomers

## Discussion

### Effects on dairy cows performance

The presence of a sorghum silage in the diet, as total replacement of corn silage, has often been associated with a decreased DMI [2, 4]. However, other authors [1, 21] did not find differences in DMI for diets based on sorghum or corn silages. The comparable DMI observed in this experiment can be partially related to the similar mean particle size of the two diets. The sorghum silage had a greater pef, peNDF, and particle size compared to the corn silage, because it was cut longer at harvesting. However, such differences were not reflected in the physical characteristics of the final diets, as the SS diet was supplemented with a greater amount of finely ground corn, allowing a reduction of particle size in the complete diet. Such a supplementation was necessary because of low starch content of sorghum, which is confirmed by others [22]. The general high DMI observed in this experiment might be related to the proper particle size of the two diets that were fractioned by sieving according to guidelines: 2 to 8% of feed material was retained on the upper screen, 30 to 50% on the middle one, and 30 to 70% on the bottom pan [23].

In this experiment milk yield was greater (+1.8 kg/d) when cows received the diet based on corn silage. Such a result is in accordance with findings of [4] and it could be due to the greater daily intake of net energy for lactation (NE_L_) when cows received the CS diet (40.1 vs. 38.9 Mcal/d, for the CS and the SS diet, respectively). However, differences disappeared when milk production was corrected for milk fat content, as the SS diet favored a + 7% increase of fat percentage compared to the CS diet. Positive effects of sorghum silage on milk fat percentage have been observed by others [2]. In the case of our study, this result could be related to the greater intake of NDF when cows received the SS diet (9.7 vs. 9.1 kg NDF per day, for cows fed the SS and the CS diet, respectively) and to the greater amount of peNDF provided by the SS diet. Performances of dairy cows were identical in terms of yield of SCM and in terms of SCM:DMI. Values of SCM and SCM:DMI found in this study were, on average, respectively 15% greater and 5% lower than those reported by [2] for cows fed diets based on brown midrib (bmr) forage sorghum or corn silages. It could be speculated that this result was mostly related to the high DMI rather than to the production level of cows that was in line with farm data.

The absence of effects on milk protein, in terms of percentage and production (kg/d), is confirmed by [1]. In the present study milk produced by cows fed the SS diet showed a protein content that was nominally greater than that produced by cows fed the CS diet. However, this effect did not have any consequence on milk urea content. Values of SCS were similar for the two diets, according to other studies [2, 3], and within the physiological range.

### Effects on milk fatty acid profile

Results of this study highlighted that milk FA profile was clearly influenced by the diet, with notable changes in proportion of the individual fatty acids. Milk was richer in PUFA when the cows were fed the CS diet, whereas the total contents of SFA and MUFA were not influenced by the dietary treatment.

The current literature encompasses several studies that evaluated effects on milk FA composition due to the replacement of corn silage with grass silages [5]. However, to our knowledge, no trial was focused on sorghum and corn silages, thus a direct comparison with literature is not possible.

Effects of corn silage on milk FA profile are well documented, but their interpretation is often complicated by the inference of chemical characteristics of corn silage and other feed ingredients included in the ration [24].

In this study, the two experimental diets differed for NDF and starch concentrations. Such a result was mainly related to the use of a forage sorghum that, as expected, had contents of NDF and starch, respectively, much greater (711 vs. 349 g NDF/kg DM) and much smaller (26 vs. 352 g starch/kg DM) compared to corn silage. In this context, inclusion of a greater amount of corn meal in the SS diet was not sufficient to compensate chemical differences between the two diets.

The addition of starch sources (i.e., cereal grains) to the ration is often associated with a decrease in milk FA with 6 to 16 carbons, and to an increase in unsaturated FA with 18 carbons [25]. Accordingly, the SS diet, being supplemented with corn meal, determined a significant decrease of C8:0, C10:0, and a numerical decrease of C12:0 and C14:0. On the contrary, the effect on 18-carbon unsaturated FA was more variable.

In addition to starch and NSC, also the dietary fat content and composition exert an influence on milk FA profile [25]. In this study, the SS diet had a lower fat content compared to the CS diet, likely because of significant supplementation with corn meal. However, both fat contents were included in ranges that are typical of rations used in dairy farms of North Italy [26]. The two diets did not differ for FA composition, with the exception of caproic acid, that was greater for the CS diet (data not shown), and stearic acid (2.48 and 2.28% on total FA, for the CS and the SS diet, respectively). Based on these data, we can speculate that differences in terms of milk FA profile between the two groups of cows were mostly related to the carbohydrate fraction rather than to the fat fraction of the diets.

Possible effects on milk FA profile due to the lactation stage, parity, or farming conditions can be excluded, because the two groups of animals were balanced for the first two parameters and farming conditions were the same for all the cows.

Despite the different effects on milk FA profile, the SS diet produced some improvements on the nutritional value of milk in comparison to CS diet. Particularly, the SS diet increased concentration of some FA with anticarcinogenic properties, such as butyric (C4:0), which is mostly protective against colon cancer, and CLA [27]. On the other hand, the CS diet increased the milk concentration of n-3 FA that also minimize the risk of cancer and cardiovascular diseases [28].

### Effects on milk coagulation properties

The role of feeding on influencing MCP is still uncertain, even if there is evidence that these properties are more influenced by genotype of animals than by phenotypic factors such as feeding [29]. However, it could be speculated that feeding and MCP are indirectly related. In this study, the protein content of milk produced by cows fed the SS diet tended to be greater than that produced by cows fed the CS diet. A recent review on modeling of MCP [30] reported that mean values (33 estimates obtained from 26 experiments) of RCT and k20 were on average 14.1 ± 4.9 min and 9.2 ± 3.1 min, respectively, for milk from Holstein-Friesian cows. Milk samples analyzed in the present study showed, on average, values of RCT and k20 included in these ranges. The same review indicated that milk produced by Holstein-Friesian cows had a mean value of a30 equal to 29.9 ± 8.0 mm. In this study, milk samples collected from cows fed the CS or SS diet revealed a mean value of a30 (35.5 mm and 31.9 mm, respectively) included in the above-mentioned range. However, the measures of a30 obtained with CRM, which was used in this study to evaluate MCP, should be considered with caution, as they were found to be less repeatable and reproducible compared to those of RCT [31].

## Conclusions

Feeding a forage sorghum silage, properly supplemented with corn meal, as total replacement of corn silage, maintained DMI, milk yield, and feed efficiency intended as the ability of cows to convert feed to milk. The substitution of corn silage with sorghum silage did not change the concentration of saturated and monounsaturated fatty acids, but reduced the concentration of polyunsaturated fatty acids in milk. In addition, n-6 and n-3 fatty acids resulted lower in milk of cows fed the sorghum diet as compare to cows fed the corn diet. Milk coagulation properties, which have a great economic relevance for the Italian dairy industry, were not altered by the substitution of the corn silage with the sorghum silage in dairy cows. These preliminary results suggest that forage sorghum silages could have a potential as substitute of corn silages in dairy cow diets.

## Abbreviations

a30: Curd firmness 30 min after the addition of rennet
ADF: Acid detergent fibre expressed inclusive of residual ash
ADL: Acid detergent lignin
CLA: Conjugated linoleic acid isomers
CP: Crude protein
CS: Corn silage
DM: Dry matter
DMI: Dry matter intake
FA: Fatty acids
k20: Time required to reach a curd firmness of 20 mm
MCP: Milk coagulation properties
MUFA: Monounsaturated fatty acids
NDF: Neutral detergent fibre assayed with a heat stable amylase and expressed inclusive of residual ash
NE_L_: Net energy per lactation
NRC: National Research Council
NSC: Non-structural carbohydrates
pef: Physical effectiveness factor
pefNDF: Physically effective NDF
PUFA: Polyunsaturated fatty acids
RCT: Rennet coagulation time
SCM: Solids corrected milk
SCS: Somatic cell score
SE: Standard error
SEM: Standard error of the mean
SFA: Saturated fatty acids
SS: Sorghum silage
TMR: Total mixed ration
